# Case report: Intravitreal methotrexate in intraocular acute lymphoblastic leukemia

**DOI:** 10.3389/fonc.2022.951362

**Published:** 2022-08-29

**Authors:** Matteo Pederzolli, Fabio Giglio, Maria Vittoria Cicinelli, Alessandro Marchese, Giulio Modorati, Sara Mastaglio, Fabio Ciceri, Francesco Bandello, Elisabetta Miserocchi

**Affiliations:** ^1^ School of Medicine, Vita-Salute San Raffaele University, Milan, Italy; ^2^ Division of Head and Neck, Ophthalmology Unit, Istituto di Ricovero e Cura a Carattere Scientifico (IRCCS) San Raffaele, Milan, Italy; ^3^ Hematology and Bone Marrow Transplantation Unit, IRCCS San Raffaele Scientific Institute, Milan, Italy

**Keywords:** intraocular leukemia, methotrexate, acute lymphoblastic leukemia, intravitreal injections, case report, intravitreal methotrexate

## Abstract

Direct leukemic infiltration of the eye is most frequently associated with acute lymphoblastic leukemia (ALL), probably due to its well-known central nervous system (CNS) tropism. Systemic treatment alone may not be sufficient for intraocular leukemia. Data on local treatment are scarce. Here, we present two cases of intraocular ALL treated with intravitreal methotrexate (MTX). Initially, anatomical improvement and visual stability were observed. The first patient experienced anatomical and visual worsening after a year of treatment. Treatment was withheld after 2 months for the second patient due to poor systemic conditions. Corneal toxicity and intraocular pressure elevation were observed in the first case. In both cases, eye involvement was associated with CNS or systemic relapse. This highlights the importance of incorporating ocular disease management in a comprehensive approach to therapy. Our experience corroborates previous findings on MTX injections as an effective and safe therapeutic option for intraocular leukemia. Further evidence is needed to consolidate the use of intravitreal MTX to treat such a debilitating localization of leukemia.

## Introduction

Ocular involvement in acute and chronic leukemia is common, and ocular symptoms may manifest at presentation or appear in later stages (1). Ocular involvement may occur as direct leukemic infiltration or secondary hematological abnormalities. Intraocular infiltration may follow or precede central nervous system (CNS) involvement. CNS localization is particularly frequent in acute lymphoblastic leukemia (ALL) (2).

The mainstay of treatment for ocular leukemia is systemic chemotherapy. Nonetheless, systemic chemotherapy drugs have scarce penetration in ocular tissues, and adjunctive local treatment is often required (3, 4). Due to the scarcity of data on intraocular treatment of leukemic infiltration, there is a high interest in reporting the outcomes of these therapies (3, 5–7). We hereby present two cases of retinal ALL infiltration treated with intravitreal methotrexate (MTX) injections.

## Case descriptions

### Case 1

The first patient was a 51-year-old woman with a history of T-ALL. At diagnosis, her white blood cell count was 300 × 10 (8)/L, and she had no CNS involvement (failed karyotype, no molecular data available). She received one cycle of induction and two consolidation courses according to the pediatric-inspired polychemotherapy scheme NILG ALL 10/07 (9); she received adequate CNS prophylaxis with intrathecal chemotherapy and 7 high-dose MTX and cytarabine as per protocol. Considering the high risk of relapse, she then received an allogeneic hematopoietic stem cell transplant (HSCT) from an HLA-identical sibling after myeloablative conditioning with busulfan and cyclophosphamide (as total body irradiation was not available). No CNS-directed therapy was given after HSCT.

**Figure 1:**
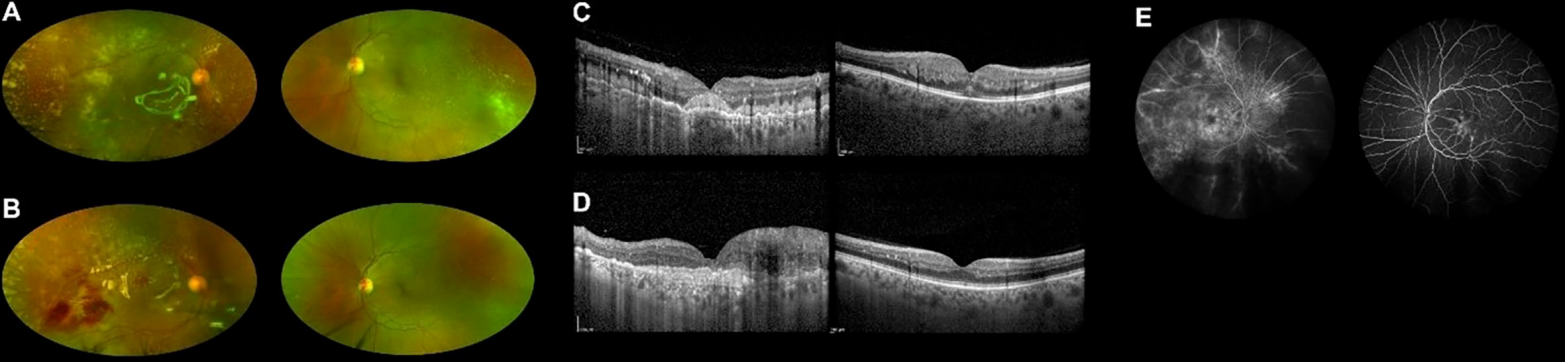
Ultra-widefield retinography of both eyes showing leukemic retinal infiltration before **(A)** and during **(B)** methotrexate treatment. OCT scans of both eyes before **(C)** and during **(D)** treatment, displaying reduced infiltration in the left eye. Fluorescein angiography at presentation showing signs of vasculitis **(E)**.

Five months after the transplant, while on complete hematologic remission with full donor chimerism, the patient lamented worsening bilateral visual loss. Best-corrected visual acuity (BCVA) was counting fingers in the right eye and 20/25 in the left eye. A combined evaluation of dilated fundus examination, ultra-widefield (UWF) retinography, and optic coherence tomography (OCT) unveiled vitritis, retinal vascular sheathing with frosted branch angiitis, and diffuse yellowish posterior-pole retinal infiltration in both eyes (**Figures 1A, C**). Fluorescein angiography confirmed bilateral retinal vasculitis of the large and small vessels (**Figure 1E**). Diagnostic vitrectomy with silicone oil was performed in the right eye: vitreous and retinal biopsies unveiled the presence of leukemic cells positive for CD34 and CD3 at immunohistochemistry. Vitreous polymerase chain reaction (PCR) analysis and culture resulted negative for viruses, bacteria, or fungi. Concurrent neurological evaluation, brain magnetic resonance imaging, and lumbar puncture were initially negative for the presence of CNS leukemic disease and graft-versus-host disease (GVHD). Since leukemia was confined to the eye, intravitreal MTX (400 μg/0.1 ml) was started in both eyes, biweekly in the first month, weekly for 2 months, and monthly thereafter [a protocol previously employed against intraocular lymphomas and leukemic infiltrates (3, 8)]. At this time, no systemic therapy was given.

During the first months of intravitreal treatment, vitreous and retinal infiltrates reduced in both eyes (**Figures 1B, D**), and visual acuity remained stable (improving to 20/20 in the left eye on one occasion). A few months later, lumbar puncture demonstrated isolated CNS recurrence of leukemia. She was treated with intrathecal methotrexate and whole-brain radiotherapy (RT) obtaining complete remission of CNS involvement. No systemic therapy was given.

Corneal toxicity presenting as superficial punctate epitheliopathy was found during treatment and was controlled with topical artificial tears and a short course of topical corticosteroids. Transient intraocular pressure (IOP) elevation (up to 40 mmHg) was noticed in the right eye and successfully managed with topical anti-glaucoma therapy. Injections were temporarily withheld.

Unfortunately, approximately 1 year after the beginning of treatment, an elevated chorioretinal mass projecting into the vitreous was observed in the right eye. CyberKnife stereotactic radiosurgery treatment was then undertaken in the right eye [anatomical outcomes are shown elsewhere (10)].

**Figure 2:**
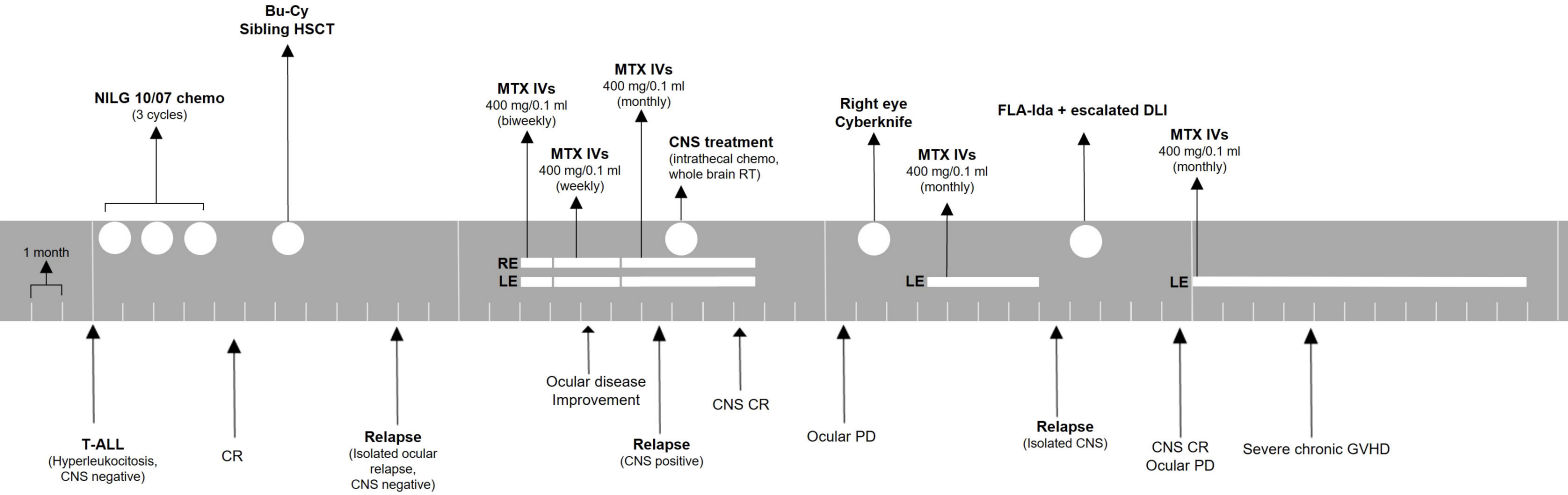
Treatment timeline for patient 1. The patient was monitored with ophthalmologic evaluations before every injection or monthly if no injection was administered. ALL, acute lymphoblastic leukemia; CNS, central nervous system; CR, complete remission; Bu-Cy, busulfan + cyclophosphamide; HSCT, hematopoietic stem cell transplant; MTX, methotrexate; IVs, intravitreal injections; RE, right eye; LE, left eye; PD, progressive disease; FLA-Ida, fludarabine, cytarabine, idarubicin; DLI, donor lymphocyte infusion; GVHD, graft-versus-host disease.

A second overt CNS relapse was documented 5 months later. The patient was treated with systemic chemotherapy with fludarabine, cytarabine, and idarubicin chemotherapy, followed by escalated dose donor lymphocyte infusion. She obtained complete CNS remission, but she suffered from progressive ocular disease. Her vision decreased to no light perception in the right eye and counting fingers in the left eye. Eventually, she developed severe chronic GVHD with skin, eye, and lung involvement, and she required immunosuppressant therapy. Ocular treatment was withheld due to poor general conditions. A timeline for patient 1 is presented in **Figure 2**.

### Case 2

The second patient was a 31-year-old man with a history of B-ALL with t(4;11)(q21;q23); *KMT2A-AFF1* rearranged. At the time of diagnosis, his white blood cell count was 175 × 10 (8)/L, and he had CNS involvement (failed karyotype, no other molecular data available). The patient obtained complete hematologic remission after pediatric inspired polychemotherapy induction (11) and four intrathecal injections of dexamethasone, MTX, and cytarabine. Persistent CNS disease was treated with high-dose cytarabine and MTX.

**Figure 3:**
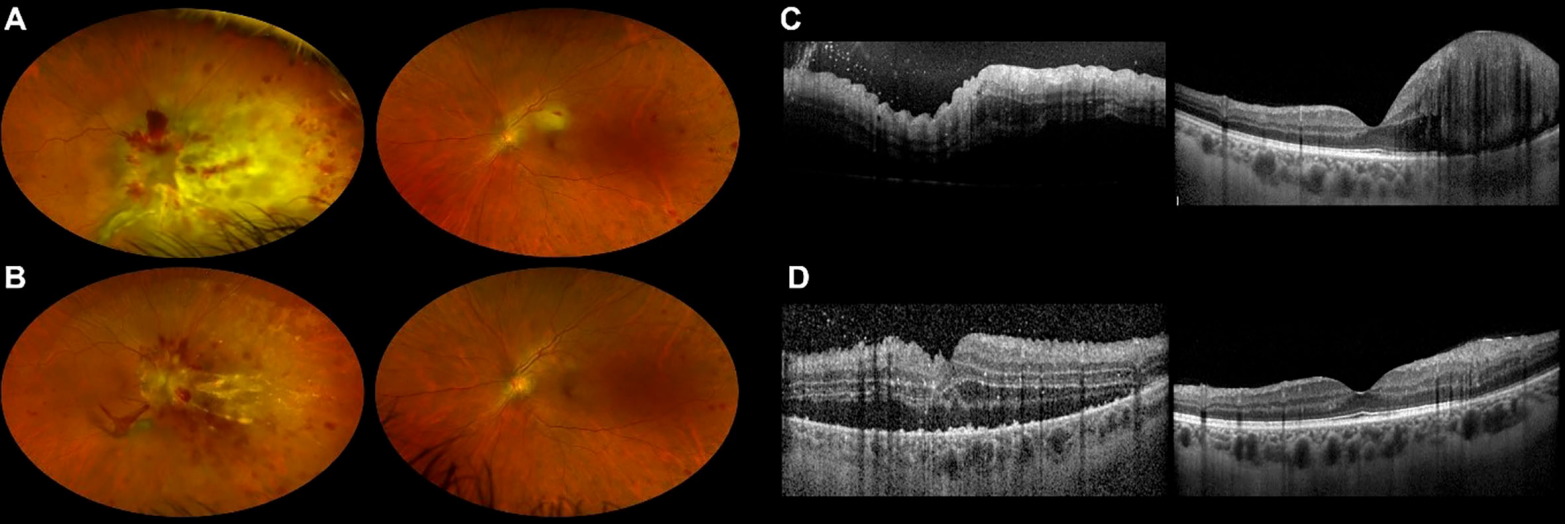
Ultra-widefield retinography of both eyes showing leukemic retinal infiltration before **(A)** and at the end **(B)** of our second patient’s methotrexate course. OCT scans of both eyes before **(C)** and at the end **(D)** of the treatment confirm morphological improvement.

Four months after diagnosis, he experienced a hematologic relapse. CNS was negative for the disease. He was treated with bispecific monoclonal antibody blinatumomab, and a transient remission was obtained. During hospitalization, the patient reported visual impairment in both eyes. BCVA at presentation was counting fingers in the right eye and 20/50 in the left eye. Dilated fundus examination, UWF retinography, and OCT collectively showed yellowish retinal infiltration with retinal hemorrhages in the posterior pole in both eyes and prominent optic disc infiltration in the right eye (**Figures 3A,C**). Aqueous humor biopsy confirmed the presence of leukemic cells, and PCR analysis excluded infectious etiologies. The patient was diagnosed with leukemic retinal infiltration. Intravitreal rituximab was not an option considering CD20 negativity on leukemic blast. We planned to put the patient on the same intravitreal MTX scheme as our first case, but his general conditions did not allow frequent injections, and we privileged the treatment of the more compromised right eye.

Despite a rarefied regimen of treatment, fundus examination, UWF retinography, and OCT showed regression of retinal infiltration in both eyes, and the patient experienced mild bilateral visual acuity improvement (**Figures 3B, D**).

**Figure 4:**
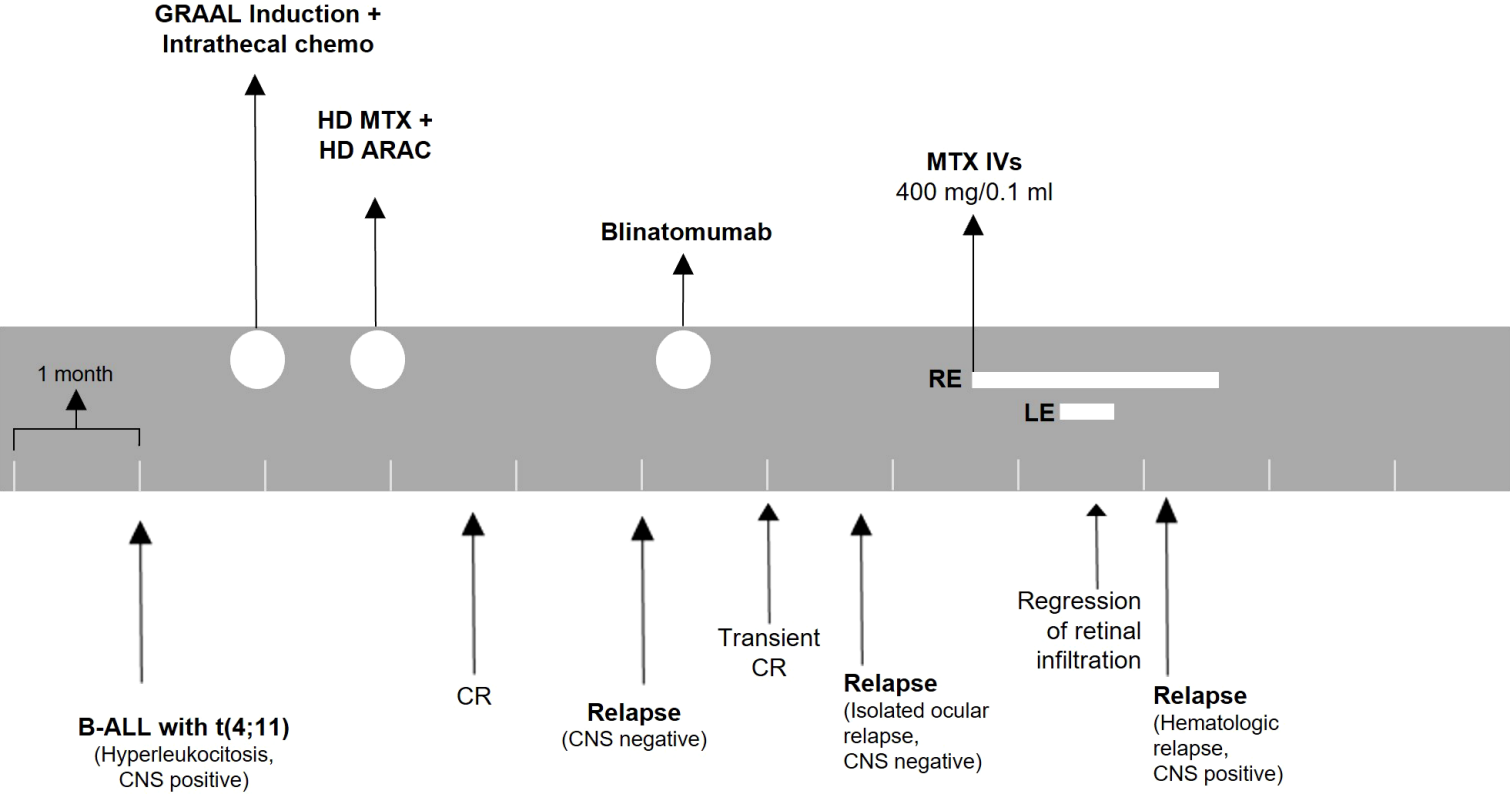
Treatment timeline for patient 2. The patient was monitored with ophthalmologic evaluations before every injection. ALL, acute lymphoblastic leukemia; CNS, central nervous system; HD, high dose; MTX, methotrexate; ARAC, cytarabine; CR, complete remission; IVs, intravitreal injections; RE, right eye; LE, left eye.

Two months after intravitreal treatment initiation, he suffered from systemic ALL relapse with CNS involvement. At this stage, the disease was unresponsive to further treatment. The patient eventually succumbed due to ALL progression and systemic complications. A timeline for patient 2 is presented in **Figure 4**.

## Discussion

CNS involvement is frequent in patients with ALL and notoriously confers a poor prognosis, while data about ocular disease are limited (2). Here, we describe two cases of ALL intraocular infiltration that occurred despite adequate CNS treatment. Both patients had high-risk leukemia. The first patient had hyperleukocytosis and had a relapse after HSCT. The second patient had hyperleukocytosis, CNS involvement, and *KMT2A-AFF1* rearrangement.

Our experience corroborates existing evidence on the local treatment of intraocular leukemia with MTX (a drug that is particularly active against ALL). Both patients benefited from MTX injections, showing reduced retinal infiltration. In our first case, MTX treatment improved funduscopic features in both eyes and, at least initially, visual function in the left eye. The second patient had a good morphologic regression of disease, even though BCVA improvement was not substantial.

Systemic chemotherapy at normal dosage has scarce efficacy on intraocular leukemic infiltration because the blood–aqueous barrier and the inner and outer blood–retinal barriers hinder the penetration of macromolecules into the ocular chambers (4).

Leukemic infiltration has been treated locally with ocular radiation and surgical vitrectomy. Dexamethasone injections have been successfully used in conjunction with pars plana vitrectomy to treat leukemic retinal and vitreous infiltrates in a 4-year-old patient with ALL: the rationale for the corticosteroid treatment was controlling inflammation and promoting apoptosis of neoplastic cells (7).

Intravitreal rituximab has been employed against intraocular lymphomas, and it would be reasonable to use it in ocular infiltration from CD20-positive leukemia (12, 13).

MTX is also employed against intraocular lymphomas (13). MTX treatment for primary leukemic invasion was first reported by Ong and White, who described a case of biopsy-proven intraocular localization of lymphocytic leukemia refractory to intravitreal triamcinolone and intrathecal MTX. Mello et al. employed intravitreal MTX for ciliary body infiltration in a case of ALL (6). Lastly, a case series by Vishnevskia-Dai et al. described the effects of intravitreal MTX treatment in 11 eyes of six patients with intraocular leukemia: signs of neoplastic infiltration and related inflammation improved with treatment, but no patient reported visual improvements (3). Previous findings on intravitreal MTX treatment for intraocular leukemic infiltration are summarized in **Table 1**.

**Table 1:** Intravitreal treatment of ocular leukemic infiltration.

Paper	Systemic disease	Number of eyes (number of patients)	Localization of infiltration	Outcomes	Side effects
Ong and White	CLL	2 (1)	Vitreous, anterior chamber, eyelids	Resolution of orbital pain and signs of infiltration and rapid VA improvement after 2 injections	None
Mello et al.	ALL	1 (1)	Iris and ciliary body	Resolution of infiltration after 8 injections, VA improvement	Keratopathy (1)
Vishnevskia-Dai et al.	ALL (7), acute promyelocytic leukemia (3), AML (1), HCL (1)^a^	11 (6)	Anterior chamber (4), vitreoretinal (8)	Regression of infiltrates, resolution of inflammation (4 patients), demise without improvement (2 patients). No VA improvement observed	Keratopathy (1)
Current series	ALL	4 (2)	Vitreoretinal	Regression of infiltrates, initial VA stabilization	Keratopathy (1), transient IOP elevation (1)

CLL, chronic lymphatic leukemia; ALL, acute lymphoblastic leukemia; VA, visual acuity; IOP, intraocular pressure.

a Data regarding treated and untreated patients.

It is also interesting to note that intravitreal MTX (together with oral valganciclovir) reduced macular edema and disc swelling in a case of leukemia-related cytomegalovirus retinitis (14). It should be noted that in both cases of our series, anatomic improvement was not strictly associated with BCVA gains, consistent with previous findings (3). Nevertheless, maintaining an acceptable visual function is a desirable outcome in the context of a comprehensive care for leukemic patients. We believe that withholding treatment would have allowed faster progression of the intraocular disease, with early irreversible visual loss.

Treatment plans need to be personalized according to individual response and the severity of ocular involvement. As our series shows, ocular disease is often associated with and may precede overt systemic and CNS disease. A close collaboration between ophthalmologists and hematologists is central to providing the holistic approach these patients need.

Injections were well tolerated by patients. Compliance was facilitated by the strong impact of visual loss on the perceived quality of life of our patients. To reduce the burden of treatment, we scheduled injections on the same day of other hospital visits whenever possible. Injections were also feasible in a severe thrombocytopenic patient like our second case (CTCAE v.5 grade 4 thrombocytopenia). The only side effects were isolated episodes of corneal epitheliopathy and IOP elevation occurring in our first patient.

We acknowledge that our study has limitations. Our series only includes two patients. However, few cases have been reported previously. The design of this paper is retrospective.

In summary, intravitreal MTX injections have proven to be an effective and safe therapeutic option in two patients with intraocular leukemic involvement, leading to reduction of retinal infiltration and stabilization of visual acuity. Further evidence is needed to evaluate the effectiveness and safety profile of intravitreal MTX in the treatment of such a debilitating localization of leukemia.

## Data availability statement

The original contributions presented in the study are included in the article/supplementary material. Further inquiries can be directed to the corresponding authors.

## Ethics statement

Ethical review and approval was not required for the study on human participants in accordance with the local legislation and institutional requirements. The patients/participants provided their written informed consent to participate in this study. Written informed consent was obtained from the individual(s) for the publication of any potentially identifiable images or data included in this article.

## Author contributions

All authors made substantial contributions in the clinical management of patients, study design, drafting or critical revision of the paper, and approved the final version of the paper.

## Conflict of interest

The authors declare that the research was conducted in the absence of any commercial or financial relationships that could be construed as a potential conflict of interest.

## Publisher’s note

All claims expressed in this article are solely those of the authors and do not necessarily represent those of their affiliated organizations, or those of the publisher, the editors and the reviewers. Any product that may be evaluated in this article, or claim that may be made by its manufacturer, is not guaranteed or endorsed by the publisher.
